# The effect of video-assisted instruction on nursing students’ skills in administering ventrogluteal intramuscular injections

**DOI:** 10.1186/s12912-025-04076-8

**Published:** 2025-11-21

**Authors:** Denizhan Yıldızbaş, Nuray Turan

**Affiliations:** 1 Faculty of Health Science, Acıbadem Mehmet Ali Aydınlar University, Kayışdağı cad. No:32 Ataşehir, İstanbul, Türkiye; 2 Department of Fundamentals of Nursing, Faculty of Nursing, Istanbul University, Süleymaniye Mah. Bozdoğan Kemer Cad. Prof. Dr. Cahit Orhan Tütengil Sok. No:1 34116, İstanbul, Türkiye

**Keywords:** Video-assisted education, Ventrogluteal injection, Psychomotor learning, Nursing education, Self-efficacy

## Abstract

**Aim:**

This study aimed to evaluate the effectiveness of video-assisted instruction as an educational intervention for improving first-year nursing students’ competence in administering intramuscular injections at the ventrogluteal site.

**Methods:**

A quasi-experimental pretest–posttest design was conducted with 106 first-year nursing students from two universities in Istanbul. A video-based training module was developed and validated by experts using the Content Validity Index, the DISCERN Inquiry Form, and the Global Quality Score. Students’ performance was evaluated before and after the intervention using a 23-item Ventrogluteal Site Intramuscular Injection Administration Checklist. Data were analyzed with descriptive statistics, McNemar’s test, and inter-rater reliability analyses.

**Results:**

Following the video-assisted training, students demonstrated a significant improvement in their procedural performance scores. Fourteen of the 23 skill items showed statistically significant gains, particularly in essential steps such as ensuring privacy, using aseptic technique, and accurate site identification. Inter-rater reliability among observers indicated good agreement, confirming the consistency of skill evaluation. These findings support the pedagogical value of standardized, multimedia-based training for novice learners.

**Conclusion:**

Video-assisted education proved to be an effective and accessible strategy for developing fundamental psychomotor skills in nursing education. Beyond enhancing specific procedural competence, this approach fosters self-efficacy, supports independent learning, and can be integrated into blended or flipped-classroom models within nursing curricula. Future studies should examine long-term skill retention and the transfer of learning to real clinical settings.

**Supplementary Information:**

The online version contains supplementary material available at 10.1186/s12912-025-04076-8.

## Introduction

One of the fundamental objectives of nursing education is not only to equip students with theoretical knowledge but also to ensure that this knowledge is safely transferred to clinical practice. In this context, medication administration constitutes an indispensable component of the nursing curriculum. Intramuscular (IM) injections, when performed with correct technique, allow rapid systemic absorption; however, incorrect administration can lead to serious complications such as abscesses, hematomas, and nerve injuries, making them among the most common parenteral drug delivery methods [1–3].

Recent studies indicate that the ventrogluteal (VG) site is the safest anatomical site for IM injections [4]. The selection of the VG site is a clinical priority for nursing education due to its superior safety profile; it is distant from major blood vessels and nerves, thereby preventing critical complications such as sciatic nerve injury, while its substantial muscle mass improves medication absorption and ensures consistent delivery [5, 6]. Despite this strong evidence, a significant ‘research-practice gap’ persists. Many nursing students and practicing nurses continue to default to the traditional dorsogluteal site, often due to a lack of knowledge, limited practical experience, or the absence of role models [7–9]. Addressing this gap by promoting evidence-based practice is essential, requiring innovative teaching approaches to instill competence and confidence in using the VG site.

To address this educational need, video-assisted education (VDE) was selected as the intervention method. VDE is an effective tool for developing psychomotor skills and motivation [10–12]. Compared to other modalities like live demonstrations or peer learning, VDE offers distinct pedagogical advantages. First, unlike traditional instructor-led demonstrations, which can vary in quality, VDE ensures high instructional standardization, presenting the correct, evidence-based technique uniformly to all students [13]. Second, VDE facilitates self-paced review, enabling students to pause, rewind, and re-watch complex steps as needed—a flexibility not afforded by live sessions [14]. This capacity for repetition and self-directed learning is crucial for enhancing procedural learning and promoting long-term knowledge retention [15].

However, video content available on digital platforms does not always adhere to scientific standards, and materials obtained from publicly accessible sources may pose risks to patient safety [16, 17]. Therefore, such content must be prepared and evaluated by reliable and authoritative sources.

This study is also grounded in Bandura’s Self-Efficacy Theory, which emphasizes that individuals’ beliefs in their ability to perform specific tasks successfully influence their motivation, learning behaviors, and performance outcomes [18]. In nursing education, self-efficacy has been identified as a key factor influencing students’ development of psychomotor skills and clinical competence [19]. Video-assisted education supports the enhancement of self-efficacy by allowing learners to observe expert demonstrations (vicarious experiences), practice skills, and gain success experiences (mastery experiences), receive constructive feedback from instructors (verbal persuasion), and reduce anxiety through a structured and supportive environment (physiological and emotional regulation) [20, 21]. Therefore, the theoretical foundation of this study suggests that video-assisted training may improve nursing students’ psychomotor performance by strengthening their self-efficacy in performing intramuscular injections in the ventrogluteal site.

This study targeted first-year nursing students as the population of interest, a decision based on two key pedagogical motivations. First, as novice learners, first-year students possess a homogeneous and minimal baseline of prior psychomotor skill, making them an ideal cohort for assessing the foundational effectiveness of an educational intervention [22, 23]. Second, introducing the correct, evidence-based technique for ventrogluteal injections before their first clinical placements is crucial. This approach aims to instill correct practice habits early and preemptively counteract the “research–practice gap,” where students might otherwise observe and adopt outdated practices—such as dorsogluteal site preference—from role models in clinical settings [4, 24].

Despite the increasing integration of technology-enhanced instructional strategies in nursing education, there remains limited evidence regarding the impact of video-assisted training on the development of psychomotor skills for intramuscular injections in the ventrogluteal site, particularly among first-year nursing students. Acquiring competence in this technique is crucial for ensuring patient safety, minimizing complications, and promoting best practices in medication administration. Addressing this knowledge gap is essential for guiding evidence-based educational interventions in nursing curricula. Therefore, this study hypothesized that video-assisted training would significantly improve first-year nursing students’ self-assessed performance scores on the Ventrogluteal Site Intramuscular Injection Administration Checklist compared to their pre-training scores. The present study aims to evaluate the effect of video-assisted training on first-year nursing students’ ability to perform intramuscular injections in the ventrogluteal site.

## Materials and methods

### Study design and participants

This study was designed as a quasi-experimental, single-group pretest-posttest design to determine the effect of video-assisted instruction on first-year nursing students’ skills in administering intramuscular injections in the ventrogluteal site.

The study population consisted of a total of 183 first-year nursing students enrolled between September 2022 and July 2023 at a state university’s Faculty of Nursing (*n* = 63) and a foundation university’s Faculty of Health Sciences, Department of Nursing (*n* = 120) in Istanbul. The final sample group, selected using a convenience sampling method, included 106 voluntarily participating students, with *n* = 46 from the state university and *n* = 60 from the foundation university. The fundamental nursing curricula regarding psychomotor skills at both institutions were confirmed to be comparable. This population was selected to ensure a consistent baseline of skill among novice learners and to instill correct practice habits before their first clinical exposure.

A post-hoc power analysis was conducted to evaluate the statistical power of the collected data. In the single-group design analysis based solely on students’ post-test results, the effect size was calculated as 0.57, indicating a moderate effect according to Cohen’s effect size classification [25]. The analysis also revealed a statistical power of 0.99, indicating a very high likelihood of obtaining significant results and a very low risk of committing a Type II error. Inclusion criteria for the study were being a first-year nursing student and regularly attending classes. Exclusion criteria included: (1) having prior clinical or simulation-based training in intramuscular injections, (2) having a visual or motor impairment that would prevent watching the video or performing the skill.

### Measurements

The study was conducted in two distinct stages. Stage 1 focused on developing and validating the educational intervention (the video) itself, ensuring its quality and reliability before use. Stage 2 then utilized this validated intervention to assess its impact on student learning outcomes. In the data collection process of the study, the first stage utilized the DISCERN Inquiry Form and the Global Quality Score (GQS), while in the second stage, the Student Information Form (SIF) and the Ventrogluteal Site Intramuscular Injection Administration Checklist (VSIIAC) were used.

#### First stage

The DISCERN Inquiry Form, developed by Charnock et al., was designed for patients and information providers to evaluate the reliability and quality of written information regarding treatment options [26]. This tool was adapted by Singh et al. for use in assessing educational videos in the health field. The form consists of five items, with each “yes” response scored as zero points, resulting in a total score ranging from 0 to 5. The average score, calculated by dividing the total score by the number of items, reflects the quality of the evaluated content. Accordingly, videos with a total score greater than three are classified as “good,” those with a score of three as “moderate,” and those with a score below three as “poor quality,” and assessments are made based on these classifications [16, 21, 27].

The Global Quality Score (GQS), developed by Bernard et al., was created to evaluate the flow, quality, information presentation, and usefulness of educational videos for individuals. This five-level scale rates videos from “1: poor quality” to “5: excellent quality.” In the assessment, the level that best reflects the quality of the video is selected [16, 21, 28].

Since all 11 experts evaluating the video were proficient in English, the original versions of the DISCERN Inquiry Form and the GQS were used without requiring cultural or linguistic adaptation.

#### Second stage

*The Student Information Form*, prepared based on the relevant literature, includes questions regarding age, gender, marital status, Grade Point Average (GPA), income level, place of residence, and employment status [29, 30]. These variables included nominal data (e.g., gender, marital status) and scale/continuous data (e.g., age, GPA). Data collected via the SIF were used solely for descriptive statistics to characterize the sample.

*The Ventrogluteal Site Intramuscular Injection Administration Checklist* was newly developed by the researchers for this study, as no single comprehensive tool was available. The 23 items were synthesized based on a comprehensive review of clinical guidelines and the relevant literature [3, 24, 31, 32]. The form was designed to assess students’ skills in administering intramuscular injections into the VG site and consists of 23 items marked with “Yes” or “No” responses. These items include four related to pre-procedure preparation, 14 related to the injection procedure, and five related to post-procedure actions. Each correctly performed step (“Yes”) was scored as 1 point and each incorrectly performed or omitted step (“No”) as 0 points. Skill proficiency was assessed based on the change in the number of correctly performed steps (i.e., ‘Yes’ responses) from pre-test to post-test for each of the 23 items individually. No total score was calculated for this analysis. This single checklist was designed to be used concurrently by both the student (as a self-assessment tool) and the independent observers (as an objective rating tool). This dual-use design allowed for a direct comparison between students’ self-perceived performance and the objective assessment by trained evaluators. To establish the reliability of the newly developed instrument, the internal consistency of the 23-item VSIIAC was assessed. The Kuder-Richardson 20 (KR-20) formula was applied to the pre-test data (n = 106), as this baseline data avoids the reduced variance and potential ‘ceiling effect’ introduced by the post-test intervention. The resulting KR-20 coefficient was 0.744, indicating an ‘acceptable’ level of internal consistency for a new instrument [33].

### Data collection

### Interventions

The study was conducted in two phases.

#### Stage 1: Preparation of the educational video

Before data collection, a scenario was developed for the Ventrogluteal Site Intramuscular Injection Training Video, and this scenario was presented to 11 faculty members from the Nursing Fundamentals department to evaluate its content validity. Necessary adjustments were made based on expert feedback, and the scenario was finalized. Content validity was calculated using the Content Validity Index (CVI) recommended in the literature [21, 34]. The video itself was designed following key instructional design principles, such as clear learning objectives, segmentation of the procedure into logical steps, expert modeling, and concise voice-over narration to minimize cognitive load. These features were informed by Cognitive Load Theory and Mayer’s Multimedia Learning Principles, which emphasize reducing extraneous cognitive processing and supporting effective visual–verbal integration to optimize learning [35, 36].

**Fig. 1:**
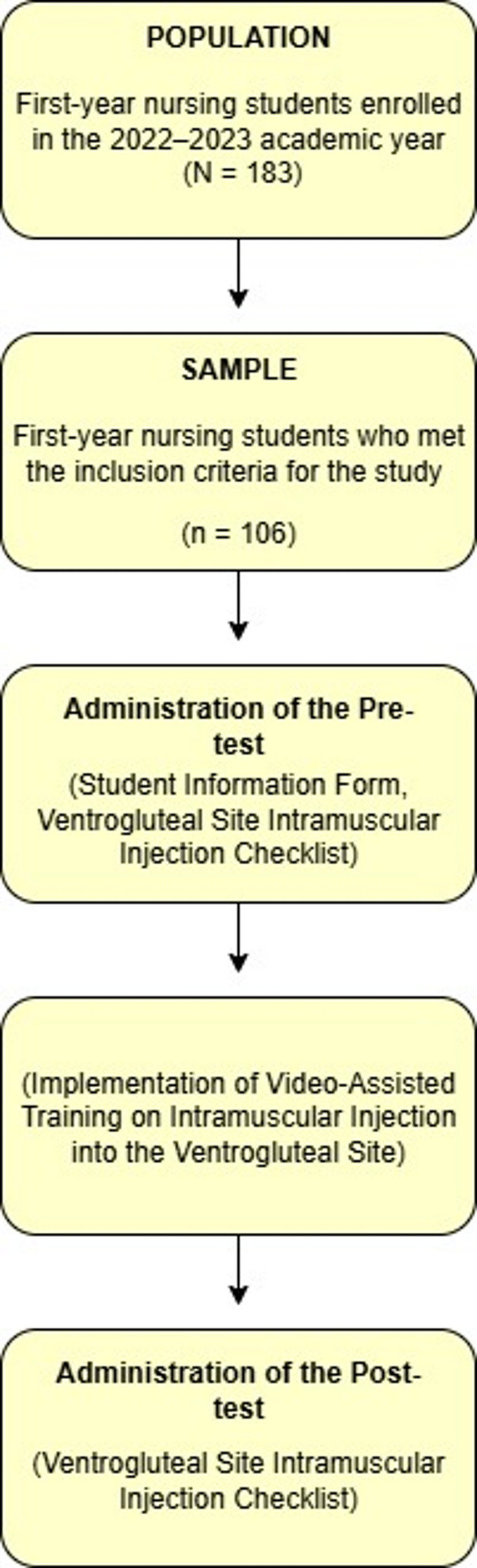
Study design

Subsequently, based on the finalized content, the procedure was performed on an IM injection simulator in a laboratory setting, and the video recording was completed [21, 31]. After recording, the video was again presented to experts, and the reliability and comprehensibility of its content were evaluated using the DISCERN Inquiry Form and the Global Quality Score (GQS). Following the finalization of the video, a pilot study was conducted with 10 nursing students; the data obtained during this stage were not included in the main study. The students were asked to watch the video and assess the reliability and comprehensibility of its content. For this purpose, the DISCERN Inquiry Form and the Global Quality Score (GQS) were completed. After the pilot study, the SIF and the VSIIAC were reviewed. Based on student feedback regarding ambiguous wording or unclear instructions, minor revisions were made to enhance their clarity and comprehensibility (Fig. 1).

#### Stage 2: Implementation of the video with students

The data collection process commenced after obtaining approval from the ethics committee and the relevant institution. The purpose of the study was explained to the nursing students included in the sample, and both written and verbal consent were obtained. The implementation was carried out according to the study design presented in Fig. 1. After being informed, the students signed the consent form, and the SIF was administered.

The evaluation process was conducted in two phases: a pre-test and a post-test.

Pre-test: Before the video intervention, students performed the procedure on a simulator and completed the VSIIAC based on their own performance. This served as the pre-test self-assessment score.

During this one-month interim period, students continued with their standard curriculum, which did not include any additional formal training or practice on intramuscular injections.

Observer Training: Prior to the post-test, the two independent faculty members (O1 and O2) assigned as observers attended half an hour training session with the primary researcher. During this session, they reviewed the 23 items of the VSIIAC, discussed scoring criteria, and evaluated sample performances to ensure inter-rater calibration.

Post-test: One month after the video intervention, students performed the procedure on a simulator again. This single performance was evaluated concurrently and independently by three parties:

The student (completing the VSIIAC as a post-test self-assessment, ‘S’).

Observer 1 (completing the VSIIAC as an objective assessment, ‘O1’).

Observer 2 (completing the VSIIAC as an objective assessment, ‘O2’).

All three raters were instructed not to communicate during the assessment. To ensure anonymity, all forms were coded. This design allowed for: (a) a pre-post comparison of students’ self-assessment scores (Table 2) and (b) an inter-rater reliability analysis between the student’s post-test self-assessment and the two observers’ post-test scores (Table 3).

### Ethical approvals

The study was conducted in accordance with the principles of the Declaration of Helsinki, after obtaining ethical approval from the Acıbadem University and Acıbadem Healthcare Institutions Medical Research Ethics Committee (Date: 22.04.2022, Number: 2022/07) and the necessary permissions from the two institutions where the research was carried out (Date: 30.05.2022, Number: 3610; Date: 30.05.2023, Number: 1780888). Nursing students were informed about the purpose, content, duration, potential benefits of the study, and the use of the collected data, and participation was based on voluntariness. Data were collected during a period when the researcher had no primary responsibilities and no teaching obligations, and written consent was obtained from the students during the face-to-face sessions.

### Data analysis

The Statistical Package for the Social Sciences (SPSS) version 26 was used for data analysis. Descriptive statistics were presented as frequency (f), percentage (%), mean, and standard deviation, where appropriate. Since the study mainly involved categorical and paired nominal data, normality assumptions for parametric testing were not required.

The reliability and comprehensibility of the educational video were evaluated using the DISCERN Inquiry Form and the Global Quality Score (GQS). Internal consistency was assessed with Cronbach’s Alpha, and inter-rater reliability was determined using the Intraclass Correlation Coefficient (ICC), both reported with their 95% confidence intervals (CIs) to indicate precision.

For the primary outcome—comparing paired binary (Yes/No) data from the VSIIAC pre-test and post-test—the McNemar test was used, which is appropriate for analyzing changes in dependent dichotomous variables. Odds ratios (OR) with 95% CIs were calculated to indicate effect size and precision [37]. This analysis is presented in Table 2.

Cohen’s kappa coefficient (κ) and Fleiss’ kappa coefficient (κ) were used to determine the agreement between the student’s post-test self-assessment (S), Observer 1’s post-test assessment (O1), and Observer 2’s post-test assessment (O2). Kappa coefficients and their 95% CIs were reported to reflect both statistical significance and the strength of inter-rater agreement [38, 39]. This analysis is presented in Table 3. For clarity, the checklist items in Tables 2 and 3 were presented with brief descriptors corresponding to key procedural steps rather than numerical item codes. All statistical tests were two-tailed, and a p-value < 0.05 was considered statistically significant.

## Results

The content validity of the 11-item educational video scenario was established by 11 nursing faculty experts. A 4-point Likert scale (e.g., 3 = ‘Quite Appropriate’, 4 = ‘Very Appropriate’) was used to rate the relevance of each item. The item-level CVI (I-CVI), calculated as the proportion of experts rating an item 3 or 4, was computed for each item. The I-CVI values ranged from 0.91 to 1.00. The scale-level CVI, using the averaging method (S-CVI/Ave), was 0.97. As all I-CVI values were well above the pre-determined acceptable level of 0.80 (Ozsaban VG,2021), the scenario was deemed to have excellent content validity. Items that received lower scores (I-CVI = 0.91), such as items 3, 4, and 8, were revised based on qualitative expert feedback to improve clarity before finalizing the video [40].

When the reliability and comprehensibility of the intramuscular injection skills training video for the ventrogluteal site were evaluated, the mean DISCERN Inquiry Form score for the video was 4.56 ± 0.60, indicating high-quality content, and the mean the Global Quality Score (GQS) was 4.70 ± 0.39, indicating that the video was useful. The Cronbach’s alpha coefficients for expert evaluations were above 0.70, showing that the expert opinions were highly consistent and reliable. Additionally, the Intraclass Correlation Coefficient (ICC) values were above 0.750, demonstrating a good level of agreement. (*p* < 0.01) (Table 1).

**Table 1:** Investigation of the reliability and comprehensibility of the training video on intramuscular injection into the ventrogluteal site

Scale	Number of Experts	Mean±SD	Cronbach’s Alpha	ICC (%95 CI)	*p*
The DISCERN Inquiry Form	10	4.56 ± 0.60	0.804	0.802 (0.492–0.949)	0.005*
The Global Quality Score	10	4.70 ± 0.39	0.750	0.762 (0.367–0.939)	**0.002**

SD: Standard Deviation, ICC: Intraclass Correlation, CI: Confidence Interval, *p < 0.01

A total of 106 first-year nursing students from both state and foundation universities participated in the study. The participants demonstrated a homogeneous distribution in terms of age, gender, marital status, and income level. The mean age of the students was 19.70 ± 1.11 years. Their academic grade point averages ranged from 1.60 to 3.72, with an overall mean of 2.67 ± 0.42.

Table 2 presents the item-based changes in student self-assessment scores from pre-test to post-test, using brief descriptors for each skill step. A statistically significant improvement (p < 0.05) was observed in 14 out of the 23 skill items following the video-assisted training. More importantly, these changes represent practically significant improvements in fundamental procedural steps. For instance, the proportion of students correctly performing ‘8. Ensures Privacy’ increased substantially from 59.4% at pre-test to 74.5% at post-test (p = 0.001). Similarly, a large gain was observed for ‘9. Dons Gloves,’ improving from 77.4% to 90.6% (p = 0.001). Other key steps like ‘7. Explains Procedure’ (66.0% to 78.3%, *p* = 0.005) and ‘2. Assesses Allergies’ (59.4% to 70.8%, *p* = 0.007) also showed statistically significant and meaningful increases. Although 9 items did not show statistically significant change, the total number of positive shifts (0→1; *n* = 318) vastly outnumbered the negative shifts (1→0; *n* = 115), indicating a clear positive overall effect of the intervention on self-assessed skill performance. (Table 2).

**Table 2:** Item-based changes in student self-assessment scores between the pre-test and post-test

Item	0→1 (No→Yes)	1→0 (Yes→No)	χ²	*p*	Result
1. Assesses Med History	12	1	9.308	0.002**	Significant (+)
2. Assesses Allergies	16	4	7.200	0.007**	Significant (+)
3. Hand Hygiene (Prep)	17	11	1.286	0.257	Not Significant
4. Prepares Supplies	12	6	2.000	0.157	Not Significant
5. Hand Hygiene (Proc)	5	2	1.286	0.257	Not Significant
6. Checks Patient ID	17	6	5.261	0.022*	Significant (+)
7. Explains Procedure	17	4	8.048	0.005**	Significant (+)
8. Ensures Privacy	19	3	11.636	0.001**	Significant (+)
9. Dons Gloves	16	2	10.889	0.001**	Significant (+)
10. Positions Patient	14	13	0.037	0.847	Not Significant
11. Palpates Site Area	11	2	6.231	0.013*	Significant (+)
12. Identifies Landmarks	6	4	0.400	0.527	Not Significant
13. Locates VG Site (Hand)	10	2	5.333	0.021*	Significant (+)
14. Injects (Angle/Speed)	15	4	6.368	0.012*	Significant (+)
15. Aspirates Correctly	9	4	1.923	0.166	Not Significant
16. Removes Needle Safely	13	4	4.765	0.029*	Significant (+)
17. Applies Pressure (No massage)	12	3	5.400	0.020*	Significant (+)
18. Ensures Patient Comfort	11	4	3.267	0.071	Not Significant
19. Disposes Needle Safely	8	3	2.273	0.132	Not Significant
20. Assesses Drug Response	26	15	2.951	0.086	Not Significant
21. Checks Site Post-Injection	20	9	4.172	0.041*	Significant (+)
22. Removes Gloves/Hand Hygiene	16	4	7.200	0.007**	Significant (+)
23. Documents Correctly	16	5	5.762	0.016*	Significant (+)

McNemar Test; total positive change (0→1) = 318; total negative change (1→0) = 115 *:*p* < 0.05; **:*p* < 0.01

The item-based agreement levels (Cohen’s κ) between the student’s post-test assessment (S) and Observer 1 (O1) and Observer 2 (O2), as well as the overall agreement among the three raters (Fleiss’ κ), are presented. According to the findings, S–O1 pairings generally demonstrated high agreement (items 7, 8, 10, 11, 14, 21, and 23), whereas S–O2 agreements mostly remained at a moderate level. O1–O2 agreement was high for some items (items 5, 6, 7, 14, and 23), but notably low for items such as 1 and 19. Non-significant agreements were observed in only a few items (particularly item 19 and partially item 12).

The overall agreement among the three raters (Fleiss’ κ = 0.611; *p* < 0.001) was at a good level, indicating a significant consensus among the evaluators (*p* < 0.01). This finding supports the reliability of the students’ post-test scores and, consequently, confirms the positive effect of video-assisted instruction on students (Table 3).

When the findings of the study are considered collectively, it was determined that the implementation process was successfully completed, the improvements demonstrated by the students following the video-assisted training were reliable, and, consequently, the video-based education contributed to the students’ learning. These results support the research hypothesis.

**Table 3:** The item-based and overall agreement coefficients between the student’s final assessment and the observers

Item	S-O1 Cohen κ	*p*	S-O2 Cohen κ	*p*	O1-O2 Cohen κ	%95 CI	Cohen’s g	*p*	Fleiss’ κ	*p*
1. Assesses Med History	0.372	< 0.001***	0.270	0.002**	0.070	[1.560, 92.290]	0.846	0.460	0.216	< 0.001***
2. Assesses Allergies	0.821	< 0.001***	0.402	< 0.001***	0.411	[1.337, 11.965]	0.600	< 0.001***	0.543	< 0.001***
3. Hand Hygiene (Prep)	0.412	< 0.001***	0.371	< 0.001***	0.532	[0.724, 3.299]	0.214	< 0.001***	0.440	< 0.001***
4. Prepares Supplies	0.428	< 0.001***	0.198	0.037*	0.605	[0.751, 5.329]	0.333	< 0.001***	0.420	< 0.001***
5. Hand Hygiene (Proc)	0.327	0.001**	0.302	0.001**	0.736	[0.485, 12.886]	0.429	< 0.001***	0.474	< 0.001***
6. Checks Patient ID	0.695	< 0.001***	0.352	< 0.001***	0.628	[1.117, 7.186]	0.478	< 0.001***	0.560	< 0.001***
7. Explains Procedure	1.000	< 0.001***	0.785	< 0.001***	0.785	[1.430, 12.631]	0.619	< 0.001***	0.855	< 0.001***
8. Ensures Privacy	0.975	< 0.001***	0.563	< 0.001***	0.592	[1.874, 21.402]	0.727	< 0.001***	0.709	< 0.001***
9. Dons Gloves	0.854	< 0.001***	0.323	0.001**	0.424	[1.839, 34.794]	0.778	< 0.001***	0.522	< 0.001***
10. Positions Patient	0.953	< 0.001***	0.413	< 0.001***	0.471	[0.506, 2.291]	0.037	< 0.001***	0.610	< 0.001***
11. Palpates Site Area	0.975	< 0.001***	0.503	< 0.001***	0.534	[1.219, 24.814]	0.692	< 0.001***	0.669	< 0.001***
12. Identifies Landmarks	0.904	< 0.001***	0.198	0.026*	0.175	[0.423, 5.316]	0.200	0.058	0.365	< 0.001***
13. Locates VG Site (Hand)	0.899	< 0.001***	0.323	0.001**	0.449	[1.096, 22.820]	0.667	< 0.001***	0.541	< 0.001***
14. Injects (Angle/Speed)	0.918	< 0.001***	0.618	< 0.001***	0.641	[1.245, 11.299]	0.579	< 0.001***	0.726	< 0.001***
15. Aspirates Correctly	0.671	< 0.001***	0.322	0.001**	0.433	[0.693, 7.306]	0.385	< 0.001***	0.472	< 0.001***
16. Removes Needle Safely	0.827	< 0.001***	0.272	0.005**	0.419	[1.060, 9.967]	0.529	< 0.001***	0.507	< 0.001***
17. Applies Pressure (No massage)	0.711	< 0.001***	0.293	0.002**	0.367	[1.129, 14.175]	0.600	< 0.001***	0.444	< 0.001***
18. Ensures Patient Comfort	0.692	< 0.001***	0.395	< 0.001***	0.659	[0.876, 8.636]	0.467	< 0.001***	0.581	< 0.001***
19. Disposes Needle Safely	0.123	0.178	-0.055	0.535	0.297	[0.707, 10.052]	0.455	0.002**	0.133	0.018*
20. Assesses Drug Response	0.784	< 0.001***	0.455	< 0.001***	0.549	[0.918, 3.272]	0.268	< 0.001***	0.596	< 0.001***
21. Checks Site Post-Injection	0.977	< 0.001***	0.579	< 0.001***	0.604	[1.012, 4.880]	0.379	< 0.001***	0.718	< 0.001***
22. Removes Gloves/Hand Hygiene	0.592	< 0.001***	0.195	0.043	0.507	[1.337, 11.965]	0.600	< 0.001***	0.428	< 0.001***
23. Documents Correctly	0.954	< 0.001***	0.777	< 0.001***	0.824	[1.172, 8.735]	0.524	< 0.001***	0.851	< 0.001***

Fleiss’ κ: 0.611; *:*p* < 0.05; **:*p* < 0.01; ***:*p* < 0.001; p-values were obtained from the z-statistics calculated for Cohen’s κ and Fleiss’ κ

## Discussion

In this study, the effect of video-assisted training on nursing students’ skills in administering intramuscular injections into the ventrogluteal site was examined using a quasi-experimental design.

The improvement observed in students’ psychomotor performance following the video-assisted training can be theoretically interpreted through Bandura’s Self-Efficacy Theory. According to Bandura, self-efficacy refers to individuals’ beliefs in their capacity to successfully perform a specific behavior, which strongly influences effort, persistence, and achievement [18]. In this study, the educational video functioned as a learning tool that enhanced students’ vicarious experiences by allowing them to observe expert modeling of correct injection techniques through both visual and auditory channels (multimodal engagement). Observation of successful role models has been shown to strengthen learners’ beliefs in their own ability to perform complex procedures [19, 41].

In addition, the video format inherently allows for repetition through self-paced review, enabling students to watch complex steps multiple times before the post-video laboratory practice. This practice then provided opportunities for mastery experiences, the most powerful source of self-efficacy, enabling students to translate observation into skill performance and experience success firsthand. Constructive instructor feedback and encouragement during these sessions served as verbal persuasion, further reinforcing confidence in their clinical abilities. The structured, low-stress environment of the simulation laboratory, likely enhanced by the students’ ability to prepare at their own pace using the video (self-paced review), also helped minimize anxiety—another factor that Bandura identified as influencing self-efficacy [20]. Collectively, these mechanisms explain why students in the post-test demonstrated both higher competence and more confident self-assessment compared with the pre-test.

The partial inconsistency observed between students’ self-assessments and observers’ ratings suggests that while task-specific self-efficacy improved, metacognitive self-evaluation skills remained underdeveloped. Previous studies similarly indicate that novice learners tend to overestimate their performance until repeated mastery experiences consolidate their sense of realistic self-efficacy [23, 42]. Therefore, incorporating reflective exercises and guided feedback within video-assisted or simulation-based programs could help align students’ perceived and actual competence levels.

Overall, these findings support the view that video-assisted training enhances nursing students’ self-efficacy and psychomotor skill acquisition. When learners believe they can perform a clinical task successfully, they are more likely to engage in practice, persist through challenges, and transfer learned skills effectively to real clinical environments—core outcomes consistent with Bandura’s theoretical model.

Regarding the intramuscular injection skills training video for the ventrogluteal site, the mean DISCERN Inquiry Form score was 4.56 ± 0.60, indicating high-quality content, and the mean and the Global Quality Score (GQS) was 4.70 ± 0.39, indicating that the video was useful. Comparisons with similar studies show that in the study by Özsaban et al., YouTube videos on ventrogluteal injections were evaluated using the DISCERN Inquiry Form and the Global Quality Score (GQS), with mean scores of 3.04 ± 1.16 and 3.71 ± 0.64, respectively [43]. Similarly, in the evaluation conducted by Özcan and Sancı on YouTube videos demonstrating robot-assisted sacrocolpopexy procedures, the mean DISCERN Inquiry Form score was 2.21 ± 1.00, and the mean Global Quality Score (GQS) was 3.04 ± 0.75 [44]. These findings indicate that the video prepared for nursing students’ training in this study is of high quality and usefulness and can be effectively used as an educational material.

It was determined that the Cronbach’s alpha value for expert opinions was above 0.70, indicating a high level of consistency and reliability among the experts. In addition, ICC values above 0.750 demonstrated a good level of agreement among expert evaluations (*p* < 0.01). In nursing education, it is well established that educational videos focusing on specific technical skills, such as ventrogluteal injections, serve as effective learning tools when structured according to expert opinions and integrated with clinical competencies. Indeed, the literature emphasizes that recording real-life scenarios and presenting them on digital platforms significantly enhances the learning process [14]. The findings of this study indicate that the video content possesses high reliability and consistency, thereby strengthening the generalizability of the results and the scientific validity of the study.

However, no statistically significant differences were found at the 5% significance level for other procedural steps, suggesting that certain sections of the training material may need to be revised and strengthened. Similarly, in the study conducted by Gürol et al., when comparing students’ correct application rates for intramuscular injection steps before and after simulation training, the success rate for the step “holding the syringe correctly” increased from 67.9% (*n* = 36) before training to 88.7% (*n* = 47) after training, with the difference reported as statistically significant (*p* < 0.001) [45]. This finding aligns with the improvements observed in the present study through video-based training. It supports the conclusion that various educational approaches can be effective in developing ventrogluteal injection skills.

### Limitations

This study has several limitations that should be acknowledged:

First, the primary analysis of effectiveness (the pre-post change reported in Table 2) was based on student self-assessment. This method carries an inherent risk of overestimation and social desirability bias. Although we included an objective observer analysis (Table 3) to measure this potential bias, our finding of ‘good’ rather than ‘excellent’ agreement (Fleiss’ κ = 0.611) confirms that the self-assessment scores must be interpreted with caution and likely represent an overestimation of true skill. Therefore, these scores cannot be seen as a direct substitute for objective evaluation by trained faculty for summative assessment. Second, the study employed a single-group, pretest-posttest design without a control group. This makes it difficult to definitively attribute all observed improvements solely to the video intervention, as factors such as the Hawthorne effect or natural maturation cannot be ruled out.

Third, the sample was drawn via convenience sampling from only two institutions, and the population sizes between these universities were unbalanced. Although the educational curricula were comparable, potential differences in participant distribution and institutional characteristics may have introduced a minor limitation regarding sample representativeness. These factors limit the generalizability of the findings to a broader population of nursing students. Fourth, the follow-up assessment was conducted at one month, which only measures short-term skill acquisition. The long-term retention of these skills was not evaluated. Fifth, an a priori sample size calculation was not performed, which is a methodological limitation. However, a post-hoc power analysis indicated that the achieved sample size (*n* = 106) provided high statistical power (0.99) to detect effects of the observed magnitude.

## Conclusion

In conclusion, this study demonstrated that video-assisted training is an effective method for enhancing first-year nursing students’ skills in administering intramuscular injections in the ventrogluteal site. The high quality and reliability of the developed educational video, confirmed by expert validation and psychometric analysis (CVI, KR-20), underscore the potential of carefully designed multimedia tools in foundational skills training. The significant improvements observed in students’ self-assessed performance, further supported by objective observer ratings, confirm the positive impact of this intervention.

This research contributes to the understanding of instructional innovation in nursing education by providing evidence for VDE as a standardized, accessible, and effective pedagogical approach, particularly for novice learners before clinical exposure. It highlights the value of integrating technology to support competence development in essential nursing procedures.

The findings of this study offer several practical implications for nursing education. The demonstrated effectiveness and high quality of the developed video suggest that VDE can be a valuable tool for teaching fundamental psychomotor skills like VG injections. Specifically, these video-based tools can be readily integrated into blended learning or ‘flipped classroom’ models, allowing students to learn foundational knowledge and observe the procedure independently before attending hands-on practice sessions. This approach can standardize instruction and potentially free up valuable faculty time for personalized feedback during skill practice. Furthermore, VDE is highly scalable for large student cohorts, overcoming logistical challenges associated with repeated live demonstrations. Finally, VDE should not be seen merely as a replacement for hands-on practice but rather as a complementary tool. It can be effectively used alongside simulation-based training or clinical practice to reinforce learning, enhance preparation, and potentially help sustain psychomotor competence over time.

While this study provides valuable insights, further research is warranted. Future studies should employ randomized controlled designs to compare the effectiveness of VDE against other modalities, such as traditional demonstration or simulation. Investigating the long-term retention of skills acquired through VDE is also crucial. Additionally, exploring the impact of VDE on students’ clinical performance with real patients, beyond simulated environments, would strengthen the evidence base. Research could also examine methods to enhance students’ metacognitive accuracy in self-assessment alongside VDE interventions.

## Supplementary Information

Below is the link to the electronic supplementary material.

Supplementary Material 1

## Data Availability

The data sets used and/or analyzed during the current study are available from the corresponding author at reasonable request.

## Abbreviations

CVI: Content Validity Index
GQS: Global Quality Score
ICC: Intraclass Correlation Coefficient
IM: Intramuscular
I-CVI: Item-level Content Validity Index
KR-20: Kuder-Richardson 20
O1: Observer 1
O2: Observer 2
OR: Odds Ratios
S: Student
S-CVI/Ave: Scale-level Content Validity Index (Averaging Method)
SIF: Student Information Form
SPSS: Statistical Package for the Social Sciences
VDE: Video-Assisted Education
VG: Ventrogluteal
VSIIAC: Ventrogluteal Site Intramuscular Injection Administration Checklist
