# Supplementary material for: The effect of video-assisted instruction on nursing students’ skills in administering ventrogluteal intramuscular injections
Source: BMC Nurs. 2025 Nov 21;24:1428. doi: 10.1186/s12912-025-04076-8 (PMC12639731; doi:10.1186/s12912-025-04076-8)
Supplement: Supplementary file 1 — Supplementary Material 1 [file 12912_2025_4076_MOESM1_ESM.docx]

| **Item No** | **Ventrogluteal Site Intramuscular Injection Administration Checklist (VSIIAC)** | **No**  **(0)** | **Yes**  **(1)** |
| --- | --- | --- | --- |
|  | Verify the individual’s medical and medication history |  |  |
|  | Assess the individual’s allergy history, including the type of allergy and the usual allergic reaction. |  |  |
|  | Perform hand hygiene. |  |  |
|  | Prepare the necessary equipment. |  |  |
|  | Perform hand hygiene again. |  |  |
|  | Verify the patient’s identity. |  |  |
|  | Explain the procedure to the patient. |  |  |
|  | Ensure patient privacy. |  |  |
|  | Don disposable gloves. |  |  |
|  | Position the patient comfortably, exposing the injection site. The most appropriate position is supine, semi-Fowler’s, or side-lying. |  |  |
|  | Palpate the injection site to check for any hardness, masses, or lesions. |  |  |
|  | Identify the injection site. |  |  |
|  | To locate the ventrogluteal site (for the left side), place the right hand on the patient’s greater trochanter, with the wrist perpendicular to the femur. The thumb points toward the patient’s groin, the index finger points toward the anterior superior iliac spine, and the middle finger is spread posteriorly as far as possible. The V-shaped area between the index and middle fingers marks the injection site. |  |  |
|  | Using the dominant hand, insert the needle quickly at a 90-degree angle into the muscle. After the needle penetrates the skin, stabilize the syringe hub with the non-dominant hand while continuing to hold the skin taut. Hold the plunger with the dominant hand without moving the syringe. |  |  |
|  | Aspirate for 5–10 seconds. If no blood appears, inject the medication at a rate of approximately 1 mL per 10 seconds. |  |  |
|  | Apply a dry cotton ball or gauze lightly over the site and withdraw the needle. |  |  |
|  | Apply gentle pressure; do not massage the site. |  |  |
|  | Assist the patient to a comfortable position. |  |  |
|  | Dispose of the needle into a sharps container. |  |  |
| 20. | Assess the patient’s response to the medication. |  |  |
| 21. | Observe the injection site for possible complications (e.g., pain, ecchymosis, induration). |  |  |
| 22. | Remove gloves, dispose of them in a medical waste bag, and perform hand hygiene. |  |  |
| 23. | Accurately document the medication administration in the nursing notes and medication chart. |  |  |
